# Some Points in the Choice of a Car—II

**Published:** 1910-11-19

**Authors:** 


### Motoring Notes.

### SOME POINTS IN THE CHOICE OF A CAR—II.

It has lately become the fashion to adopt the worm, instead of the bevel, for the final drive, and this is quite efficient when adequate lubrication is provided. It is worth noting that the chain-drive has now been practically abandoned, except for commercial vehicles and special purposes.

Ignition by the high-tension magneto or by the dual system—magneto, supplemented by coil and accumulator—are the only forms now to be seen. The dual system is desirable, since it is one that is at times of considerable utility; though, in fact, the reliability of the modern magneto is such that an additional resource is seldom necessary. The steering column should be provided with a good rake—the old-fashioned style with upright steering pillar being a sign neither of efficiency nor of smartness, and a smart-looking raked pillar is an aid to selling a car later on. At the same time it should be seen that the rake is not such as to necessitate a few extra inches in the length of the legs, with which the purchaser has not been provided by Nature—in other words, while the body is in a free and comfortable position over the steering wheel the feet should easily be able to reach the control pedals. The spark advance and throttle levers should be on either the steering wheel or the column, but an accelerator pedal is essential to efficient control, and, wherever possible, both hand and foot control of the throttle should be insisted upon.

### Brakes.

In the matter of braking, the pedal brake on the back-wheel hubs is undoubtedly the best principle, and one that relieves the transmission shaft of much unnecessary strain. Experience, however, goes to show that in the case of the latter type repairs and replacements are apt to be more expensive than in other types. In the great majority of cases the purchaser will have no choice, since the usual practice is to fit a pedal brake on the transmission shaft and a hand brake acting on the back wheels. Front-wheel brakes acting on the Allen-Liversidge principle can easily be fitted, and should be insisted upon, being very efficient in practice and definite preventers and correctors of skidding and side-slip.

Careful attention should be given to the springing, and cars of medium power should have long and flexible springs. The three-quarter elliptical spring is fairly efficient, but the interposition of a coil or lever spring between the leaf spring and the frame makes a wonderful difference. The ideal is a system of pneumatic suspension, but, unfortunately, no practical method of securing this has yet been attained. It is worth remembering that spring shackles require lubrication, and should be provided with holes for this purpose, and adequately provided with dust caps.

The undershield is a minor matter, but one that adds much to the comfort of the owner and the convenience of other users of the road; it is a great factor in the silent running of a car, while it is useful in keeping out dirt from the running mechanism, and serves largely to keep down the dust nuisance. The two essentials are that it should reach as far forward towards the radiator as possible, and that it should be properly fitted. Shackle or clip fastenings allow of more easy removal than bolts and nuts, but they must fasten properly.

Bodies are a matter of taste, the purse, and the purpose for which the car is intended. For all-round purposes the open touring body is best, with a Cape-cart hood in reserve for bad weather. But whatever type is selected, care should be taken to secure adequate leg room inside, without which comfort is impossible. The high flush-sided "torpedo" body is generally less comfortable and roomy inside than its outward appearance would suggest. Doors should be fitted to the front seats in any case, and they should be as high as possible. Consideration should always be given to the relation of weight to horse-power, as it would be absurd to expect a low-powered car to perform well with a body of disproportionate weight.

### Expenses.

In running expenses the heaviest item will be that of tyres. In the absence of a really satisfactory tyre that can neither burst nor puncture, recourse must be had to the ordinary type of pneumatic as the only thing possible. For a car of 10 to 15 h.p. the most efficient size of tyre will be 815 by 105. The 810 by 90 will fit the same size of rim, but will give far less resiliency and ease of movement—in fact, one should always fit the largest sized tyre the wheel rims will take.

One should use only the best in accessories, as the most expensive car may easily be spoilt in appearance by the use of inferior accessories. The wind screen is a case in point. The various points that should be looked for are: that the dashboard is strong enough to bear the screen; that the latter is in harmony with the contour of the dash; that the break is not immediately in front of the eyes; and that, when folded, the screen does not interfere with the opening of the bonnet.

Every speck of dirt and dust that can be kept off the chassis of the body is so much gain to the motorist, particularly if he drives his own car, and the mudguards are therefore of the utmost importance. In this connection the dome-shaped type, known as the "Frankonia," are specially to be recommended.

Head lamps should be either acetylene or electric—the latter, perhaps, involving less care and attention to keep clean and in good order.

These are the main points to be observed in the selection of a car, and it is hoped that they may be of some assistance to the prospective purchaser in securing satisfaction.

